# Reproducibility of an Automated Quantitative MRI Assessment of Low-Grade Knee Articular Cartilage Lesions

**DOI:** 10.1177/1947603520961165

**Published:** 2020-09-29

**Authors:** Vladimir Juras, Pavol Szomolanyi, Markus M. Schreiner, Karin Unterberger, Andrea Kurekova, Benedikt Hager, Didier Laurent, Esther Raithel, Heiko Meyer, Siegfried Trattnig

**Affiliations:** 1 High-Field MR Centre, Department of Biomedical Imaging and Image-Guided Therapy, Medical University of Vienna, Vienna, Austria; 2 Institute of Measurement Science, Slovak Academy of Sciences, Bratislava, Slovakia; 3 Department of Orthopedics and Trauma Surgery, Medical University of Vienna, Vienna, Austria; 4 CD Laboratory for Clinical Molecular MR Imaging, Vienna, Austria; 5 Novartis Institutes for Biomedical Research, Department of Translational Medicine, Basel, Switzerland; 6 Siemens Healthcare GmbH, Erlangen, Germany; 7 Austrian Cluster for Tissue Regeneration, Vienna, Austria

**Keywords:** cartilage repair, repair, magnetic resonance imaging, diagnostics, knee, joint involved, osteoarthritis, diagnosis

## Abstract

**Objective:**

The goal of this study was to assess the reproducibility of an automated knee cartilage segmentation of 21 cartilage regions with a model-based algorithm and to compare the results with manual segmentation.

**Design:**

Thirteen patients with low-grade femoral cartilage defects were included in the study and were scanned twice on a 7-T magnetic resonance imaging (MRI) scanner 8 days apart. A 3-dimensional double-echo steady-state (3D-DESS) sequence was used to acquire MR images for automated cartilage segmentation, and T2-mapping was performed using a 3D triple-echo steady-state (3D-TESS) sequence. Cartilage volume, thickness, and T2 and texture features were automatically extracted from each knee for each of the 21 subregions. DESS was used for manual cartilage segmentation and compared with automated segmentation using the Dice coefficient. The reproducibility of each variable was expressed using standard error of measurement (SEM) and smallest detectable change (SDC).

**Results:**

The Dice coefficient for the similarity between manual and automated segmentation ranged from 0.83 to 0.88 in different cartilage regions. Test-retest analysis of automated cartilage segmentation and automated quantitative parameter extraction revealed excellent reproducibility for volume measurement (mean SDC for all subregions of 85.6 mm^3^), for thickness detection (SDC = 0.16 mm) and also for T2 values (SDC = 2.38 ms) and most gray-level co-occurrence matrix features (SDC = 0.1 a.u.).

**Conclusions:**

The proposed technique of automated knee cartilage evaluation based on the segmentation of 3D MR images and correlation with T2 mapping provides highly reproducible results and significantly reduces the segmentation effort required for the analysis of knee articular cartilage in longitudinal studies.

## Introduction

Magnetic resonance imaging (MRI) is a valuable tool that provides the ability to detect signs of osteoarthritis (OA) in the whole joint and in all joint structures, as well as to quantify changes in cartilage volume and thickness during the course of the disease.^1,2^ Recently, a number of MR methods have been developed that are relatively specific for the proteoglycan and collagen content in OA-affected articular cartilage. These compositional markers can noninvasively determine collagen content and organization,^3^ proteoglycan content,^4^ biomechanical properties,^5^ and also detect early-stage focal cartilage lesions.^6^ Transverse relaxation time (T2) mapping is a well-established quantitative MRI method, which reflects the interplay of water content and collagen matrix organization.^7,8^ The anisotropy of cartilage tissue results in T2 variation from deep to superficial cartilage layers, depending on the collagen fiber orientation.^9^ T2-mapping is often used in longitudinal studies where it can provide valuable information on collagen matrix status as the disease progresses.^10,11^ On ultra-high-field MR scanners, more progressive sequences for T2-mapping can be used rather than a conventional multi-echo spin-echo sequence, such as triple-echo steady state (TESS) sequence, which provide 3-dimensional (3D) knee coverage, lower specific absorption rate demands, and shorter measurement times.^12-14^ Additionally, texture analysis of quantitative MR maps using gray-level co-occurrence matrix (GLCM) features provides additional information on collagen organization and can be used to determine cartilage status.^15^

Cartilage and bone deformations and cartilage thinning can be manually quantified using high-resolution morphological MRI. However, if this approach is carried out manually, it requires an enormous amount of time and manpower and may be subject to relatively high inter-/intrareader variability. Recently, many techniques for automated cartilage segmentation have been introduced, including intensity- and edge-detection-based^16,17^ approaches, clustering,^18^ deformable models,^19^ and atlas-/graph-based methods.^20^ Fripp *et al*.^21^ designed a segmentation scheme that involves the automated segmentation of bones using a 3D active shape model, the extraction of the expected bone-cartilage interface (BCI), and cartilage segmentation from the BCI using a deformable model that utilizes localization, patient-specific tissue estimation, and a model of the thickness variation.

The logical next step for automated cartilage segmentation is the application to quantitative MR cartilage evaluation. This can be a tedious task when performed manually. Hesper *et al*.^22^ presented a reader-independent automated hip cartilage segmentation for delayed gadolinium-enhanced MRI of cartilage (dGEMRIC) for the assessment of biochemical cartilage status. Norman *et al*.^23^ developed a convolutional neural network (CNN)–based method for automated T1ρ evaluation, demonstrating the ability to quantify relaxometry and morphology in a single session. All the aforementioned methods, however, used a bulk cartilage segmentation and a quantitative assessment. For analysis of cartilage affected by OA, it is important to quantify any alterations in cartilage subregions, as they can be affected differently.^24^ In particular, weightbearing and nonweightbearing regions have different cartilage composition and function, and also cartilage layers change differently during the course of OA progression.^25,26^

Therefore, the goals of this study were (1) to assess the reproducibility of an automated knee cartilage segmentation with a model-based algorithm in 21 cartilage regions each with 3 layers, (2) to develop and validate a coregistration approach of DESS images and TESS T2 maps, and (3) to compare the results with manual segmentation.

## Materials and Methods

### Patient Cohort

This was a single-center prospective study and was approved by the institutional review board (The Ethics Committee of the Medical University of Vienna No. 1978/2014), and all participants provided written informed consent. Thirteen patients with a femoral cartilage defect of ICRS (International Cartilage Repair Society) grade I in the lateral or medial femoral condyle with (6 females, mean age ± standard deviation: 50.8 ± 4.4 years, and 7 males: 50.2 ± 6.1 years) were involved in the study. Cartilage lesion ICRS grade I was defined as cartilage with a normal thickness and a normal smooth surface, but with intrachondral signal alterations. Inclusion criteria comprised ICRS grade I cartilage lesions in the femoral condyle and risk factors for cartilage disease progression, such as the presence of an anterior cruciate ligament or meniscal tear. Subjects with contraindications to MRI, such as pacemakers, implants, or pregnant subjects, were excluded from the study.

### MRI Protocol

All subjects underwent an MR examination on a whole-body investigational 7-T MR scanner (Siemens Healthineers, Erlangen, Germany) with a dedicated 28-channel knee coil (Quality Electrodynamics, Mayfield Village, OH, USA). A 3D double-echo steady-state sequence (3D-DESS) was used to acquire high-resolution MR images for automated cartilage segmentation. T2-mapping was performed using 3D triple-echo steady-state (3D-TESS).^15^ The T2 maps were reconstructed online on the scanner using an IceLuva script.^30^ All sequence parameters are listed in **Table 1**. To analyze test-retest variability, the measurements were repeated twice: at baseline and after 8 days. In addition to the mean T2 values, each region of interest (ROI) was evaluated using texture analysis with a GLCM.^27,28^ Based on the literature research and in-house optimization, the following parameters were used: direction 90° (parallel to cartilage surface); 16 levels of gray; and an offset of 1. All slices of each cartilage region were analyzed and averaged. Each ROI was preprocessed by rotation, flattening, and resampling. Using the MatLab library,^29^ from a total of 23 features, the 7 most suitable for cartilage assessment were selected: autocorrelation, contrast, correlation, dissimilarity, energy, entropy, and homogeneity.

**Table 1.** Image Acquisition Parameters for Morphological (3D-DESS) and Quantitative (3D-TESS T2-Mapping) Analysis.

Sequence Parameters	3D-DESS	3D-TESS for T2-Mapping
Image plane	Sagittal	Sagittal
Slice thickness	0.5 mm	3 mm
Slice spacing	0.5 mm	3 mm
Repetition time	8.86 ms	9.76 ms
Echo time	2.55 ms	5.1 ms
Averages	1	1
Acquisition matrix	320 × 320	384 × 346
Field-of-view	160 × 160 mm^2^	143 × 143 mm^2^
Flip angle	18°	15°
Total acquisition time	3:57 min	3:48 min
Pixel bandwidth	347 Hz/px	501 Hz/px

3D-DESS = 3-dimensional double-echo steady-state; 3D-TESS = 3-dimensional triple-echo steady-state

### Manual Cartilage Segmentation

All 3D-DESS images were segmented manually by a medical student (K.R.) and supervised by an orthopedic surgeon with extensive experience in musculoskeletal imaging (M.S.), who also edited the automated segmentation, if necessary. Manual segmentation was done only for bulk femoral cartilage, bulk patellar cartilage, and lateral and medial tibial cartilage, rather than for all 21 subregions separately, since matching the exact perimeters of these subregions manually is difficult and even a slight mismatch might introduce significant bias. The corresponding regions from the automated segmentation were concatenated. The ability of the algorithm to reproducibly segment the subdivision into 21 subregions was demonstrated with the test-retest assessment.

### Automated Cartilage Segmentation

Knee articular cartilage was segmented using the prototype MRChondralHealth software (version 2.1, Siemens Healthcare, Erlangen, Germany), which is a model-based segmentation algorithm. The basic scheme consists of 4 stages: preprocessing, atlas alignment, bone segmentation, and cartilage segmentation.^21,30^ According to anatomical landmarks introduced by Surowiec *et al*.,^31^ knee cartilage was divided into 6 patellar, 6 tibial, and 9 femoral subfields. Each segment was further divided into 3 layers defined as three thirds along the surface-BCI axis.

After the cartilage was segmented, the resulting files (21 cartilage subfields, thickness map, layer definitions, and bone segmentation) were converted from the image format *mlimage* to the *nifti* format. All files were further processed using MATLAB scripts.

### Morphological and Quantitative Image Registrations

To coregister T2 maps with morphological 3D-DESS images, an algorithm developed in MATLAB (version 2019b, The MathWorks, Inc, Natick, MA, USA) was used. First, the matching slices of 3D-DESS and 3D-TESS (second echo) were identified using the DICOM (digital imaging and communications in medicine) header information (slice location and patient orientation). Then, a multimodal coregistration method was applied using spatial mapping of fixed images (DESS) and moving images (TESS). Affine transformation with 12 degrees of freedom was used. Optimizer function parameters were determined by a previous iterative process, while a similarity index map was used as a quantitative coregistration quality marker.^32^ The resultant optimizer parameters were as follows: initial radius = 0.001; epsilon = 1.5e-4; growth factor = 1.01; and maximum iterations = 300. Finally, the resulting transformation was applied to the actual T2 map. The T2 map was further preprocessed by thresholding values lower than 5 ms and higher than 150 ms.

### Data Evaluation

Results from the automated segmentation (A) were compared with manual segmentation (M) sets from five regions (patella, lateral tibia, medial tibia, femur, and all regions combined). Three measures were used: the Jaccard coefficient ((the number of voxels in A+M) / (the number of voxels in either A or M)) and the Dice coefficient (2 * |A| ∩ |M| / (|A| + |M|). A Jaccard coefficient higher than 0.7 and a Dice coefficient higher that 0.80 were considered acceptable.

To perform a test-retest of automated cartilage segmentation and automated quantitative parameter extractions, the data from all patients were assessed independently from baseline scans and the follow-up scan after 8 days. Extracted features (cartilage volume, thickness, T2 values, and GLCM features) from both time points were compared using the standard error of measurement (SEM) and smallest detectable change (SDC).

To validate automatically extracted T2 values from the knee segments, T2 maps of 5 knees were segmented manually, selecting 21 regions corresponding to automated segmentation. The absolute difference of T2 values in milliseconds, and the relative difference in percentage and volume difference was calculated in bulk for each of the segments, as well as in 3 cartilage layers (cartilage divided into equal three thirds along the superficial-deep axis).

To validate the ability of automatically extracted parameters to detect low-grade cartilage lesions, the location of each lesion was determined by a radiologist with 25 years of experience (S.T.). The Student paired *t* test was used to find the difference in the means of all variables in cartilage segments containing a lesion and in cartilage lesion-free segments. A *P* value lower than 0.05 was considered statistically significant.

## Results

**Figure 1.**
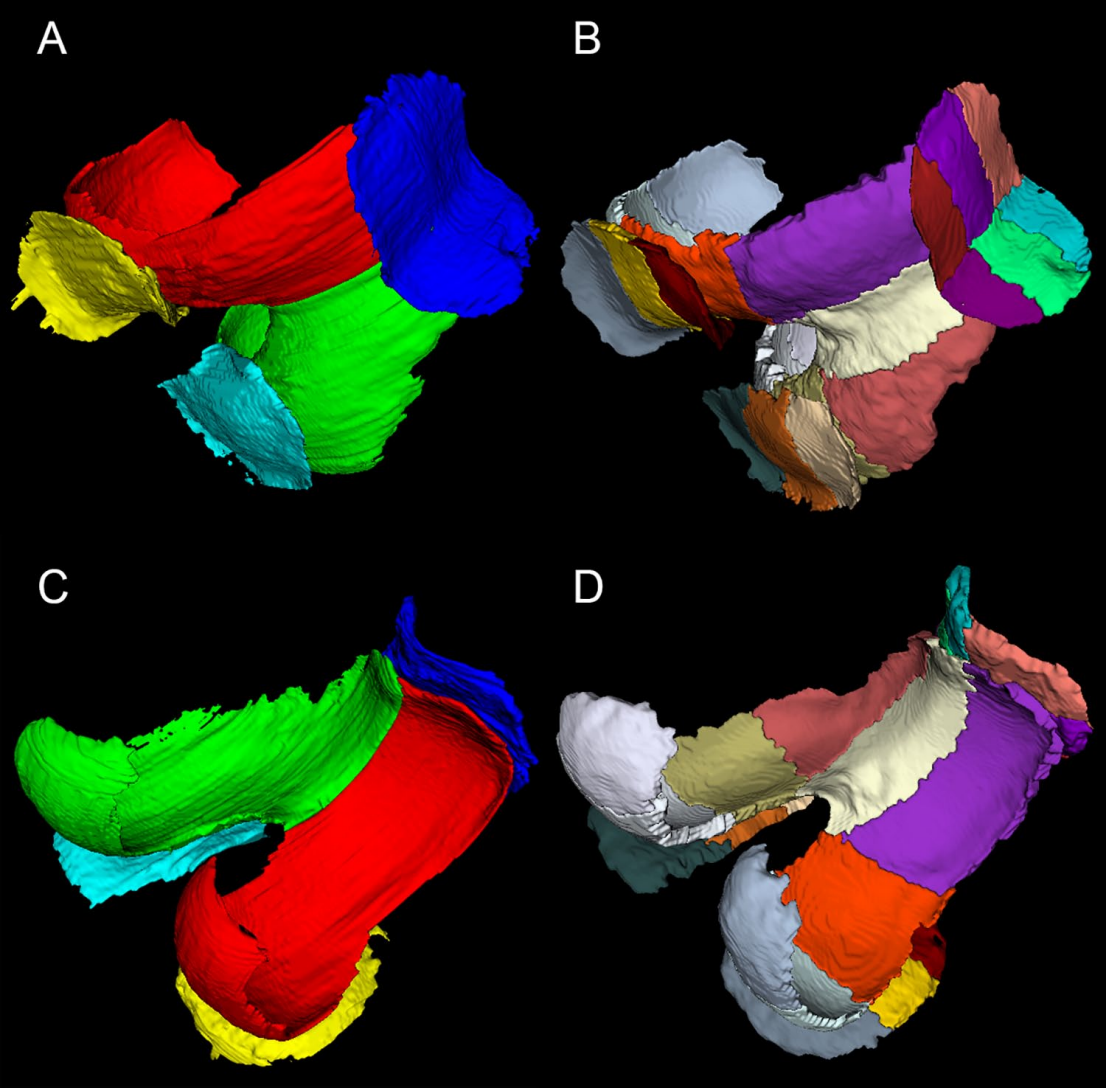
An example of manual and automated cartilage segmentation: (**A**) manual segmentation caudal view; (**B**) automated segmentation caudal view; (**C**) manual segmentation cranial view; and (**D**) automated segmentation cranial view.

**Figure 2.**
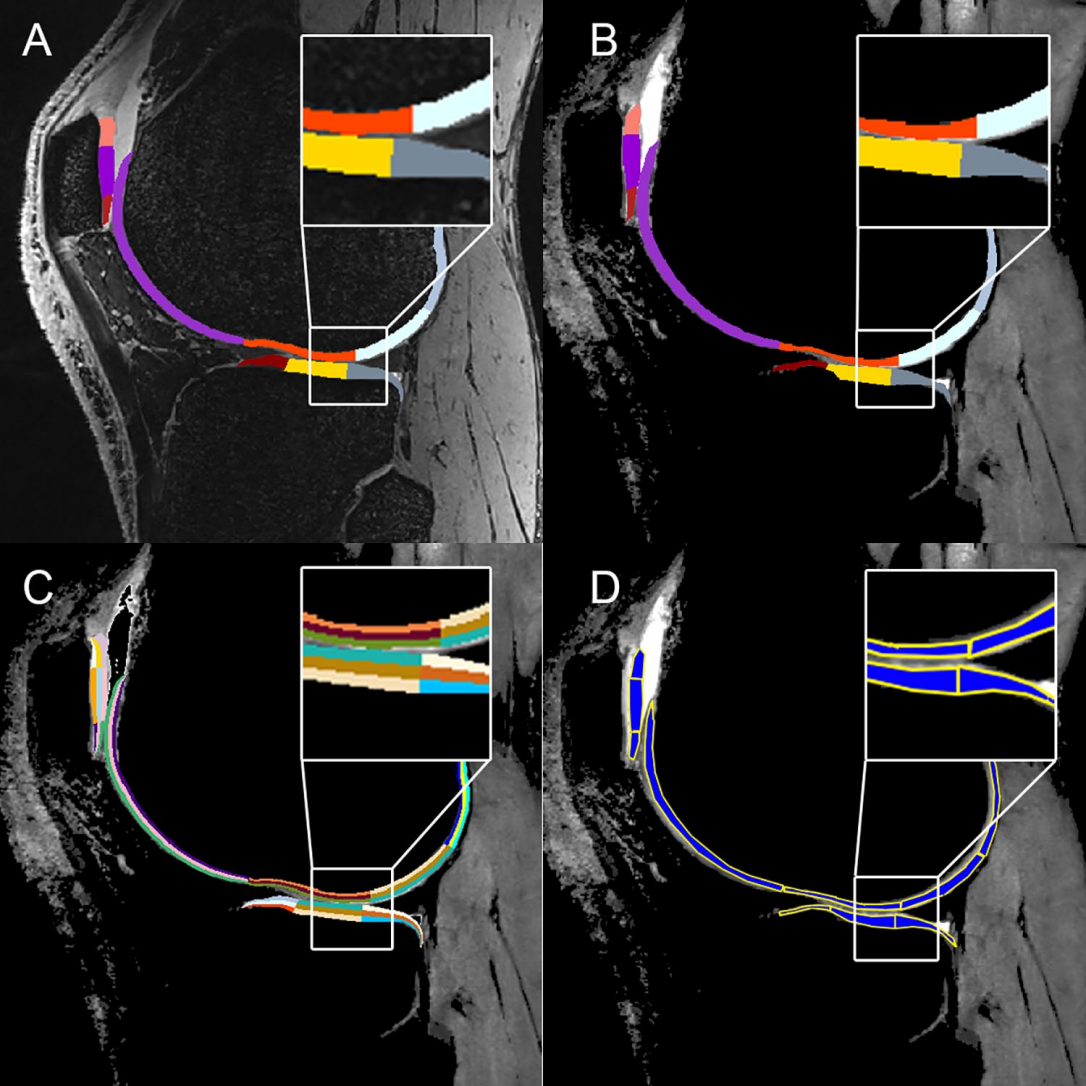
(**A**) Sagittal view of a knee overlaid with the automated cartilage segmentation; (**B**) coregistered T2 map overlaid with the automated cartilage segmentation; (**C**) coregistered T2 map overlaid with the automated cartilage segmentation of layers; and (**D**) manual segmentation of the coregistered T2 map.

The mean segmentation time for automated segmentation was 8.2 ± 2.0 minutes per case, and for manual segmentation, ~7 hours per case. The postediting of automated segmentation took ~20 minutes per case. Typically, small corrections were needed in all cases, most often in the lateral posterior femur, and the anterior and posterior lateral tibia. The exemplary manual and automated segmentations in various views are depicted in **Fig. 1** and **2**. The similarity coefficients between manual and automated segmentation ranged from 0.7 to 0.722 and from 0.825 to 0.882 for the Jaccard coefficient and the Dice coefficient, respectively. In case of postedited automated segmentation, the similarity to manual segmentation ranged from 0.788 to 0.845, from 0.828 to 0.895 for the Jaccard coefficient and the Dice coefficient, respectively. All coefficients are listed in **Table 2**.

**Table 2.** The Comparison of Automated and Manual Cartilage Segmentation Expressed by the Jaccard Coefficient and the Dice Coefficient (Both Fully Automated and Automated with Postediting Options Are Listed).

Cartilage Region	Fully Automated		Fully Automated with Postediting	
	Jaccard	Dice	Jaccard	Dice
Patella	0.706	0.855	0.823	0.879
Lateral tibia	0.700	0.850	0.788	0.861
Medial tibia	0.702	0.825	0.832	0.828
Femur	0.722	0.882	0.845	0.895
All regions combined	0.710	0.834	0.822	0.866

**Figure 3.**
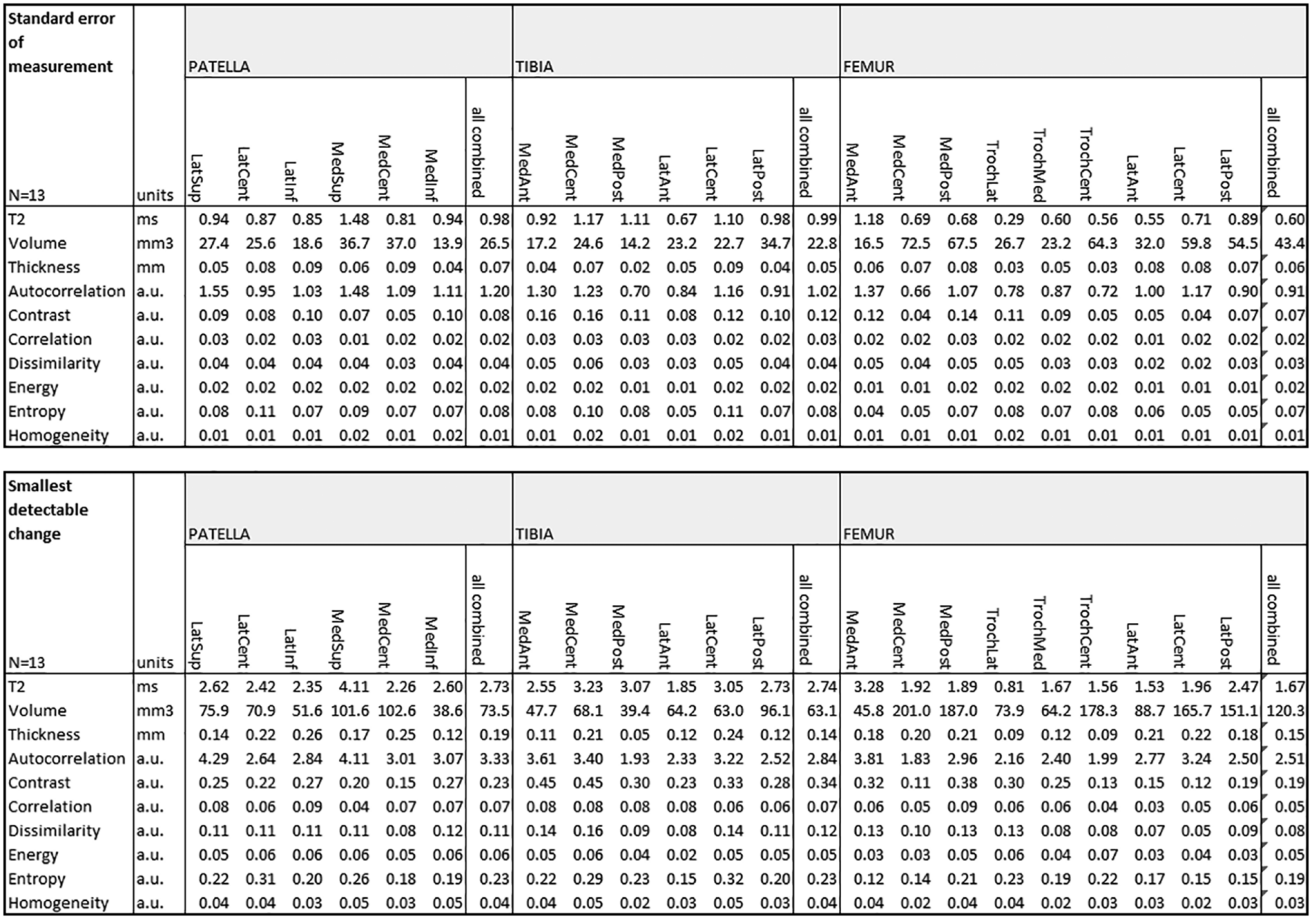
Test-retest of automated cartilage segmentation and automated quantitative parameter extractions from baseline scan and repeated scan after 8 days.

Test-retest analysis of automated cartilage segmentation and automated quantitative parameter extractions revealed excellent reproducibility, especially in femoral cartilage for T2, volume, and thickness detection, mean SDC was 1.97 ms, 120.3 mm^3^, and 0.15 mm, respectively. Relatively small SDC was found also for GLCM features. All SEM and SDC parameters are listed in **Fig. 3**.

The comparison of automated and manual T2 evaluation showed relatively high agreement. In case of bulk T2 values, the mean difference of T2s in all subregions was 4.26 ± 1.22 ms (3.55%), while the highest agreement was found in the tibia (3.11 ± 0.81 ms, 2.74%), and the lowest in the femur (5.89 ± 3.44 ms, 6.21%). The overall difference between manual and automated segmentation measures of T2 in the different zones was as follows: in the superficial zone, 1.57 ± 0.91 (6.26%); in the transitional zone, 1.82 ± 1.11 ms (6.25%); and in the deep zone, 1.49 ± 1.13 ms (4.85%). All T2 differences between manual and automated evaluation are listed in **Table 3**.

**Table 3.** The Differences in T2 Values Calculated from Automated and Manual Evaluations in 3 Layers.

	Region	Layer	ΔT_2_ (%)	ΔT2 (ms)	Δvoxels
Patellar cartilage	Lateral superior	Deep	7.81	1.77	169
		Transitional	11.85	2.91	−267
		Superficial	−3.10	−0.65	465
	Lateral central	Deep	7.22	1.66	24
		Transitional	−7.67	−2.39	−40
		Superficial	9.56	3.49	93
	Lateral inferior	Deep	−11.06	−2.62	−50
		Transitional	4.87	1.36	74
		Superficial	3.74	1.16	−56
	Medial superior	Deep	1.98	0.60	−262
		Transitional	−3.00	−1.00	440
		Superficial	0.63	0.23	874
	Medial central	Deep	12.92	3.73	317
		Transitional	2.68	1.08	−332
		Superficial	2.77	1.17	2884
	Medial inferior	Deep	−2.48	−0.65	−678
		Transitional	7.39	2.15	1052
		Superficial	2.59	0.81	1291
Tibial cartilage	Medial anterior	Deep	−8.18	−1.50	247
		Transitional	12.23	2.90	267
		Superficial	13.44	3.28	96
	Medial central	Deep	−3.63	−0.84	−957
		Transitional	11.89	3.10	477
		Superficial	6.47	1.70	74
	Medial posterior	Deep	−5.18	−1.90	−1153
		Transitional	−1.31	−0.40	2124
		Superficial	0.39	0.10	2370
	Lateral anterior	Deep	1.64	0.35	−211
		Transitional	11.73	3.13	−574
		Superficial	−6.80	−1.98	310
	Lateral central	Deep	13.62	2.97	918
		Transitional	0.57	0.17	231
		Superficial	−6.36	−1.90	−10
	Lateral posterior	Deep	−6.34	−1.64	127
		Transitional	9.54	3.32	52
		Superficial	5.66	1.72	−366
Femoral cartilage	Medial anterior	Deep	3.00	0.68	531
		Transitional	4.70	1.39	−433
		Superficial	−4.49	−1.44	204
	Medial central	Deep	1.22	0.41	67
		Transitional	4.32	1.70	164
		Superficial	2.03	0.79	−568
	Medial posterior	Deep	−1.38	−0.53	745
		Transitional	9.08	3.48	1394
		Superficial	−1.46	−0.51	−1930
	Trochlear lateral	Deep	9.26	2.26	560
		Transitional	0.55	0.16	−1679
		Superficial	3.02	0.92	2126
	Trochlear medial	Deep	−7.72	−1.69	1174
		Transitional	−6.33	−1.58	−1891
		Superficial	5.85	1.54	2066
	Region	Layer	ΔT_2_ (%)	ΔT2 (ms)	Δvoxels
	Trochlear central	Deep	7.74	1.91	1244
		Transitional	−3.99	−1.26	−1471
		Superficial	10.53	3.28	1140
	Lateral anterior	Deep	6.21	1.03	921
		Transitional	9.93	2.35	−962
		Superficial	−1.12	−0.37	229
	Lateral central	Deep	−11.19	−2.95	−96
		Transitional	0.37	0.14	89
		Superficial	10.11	3.87	−175
	Lateral posterior	Deep	−9.28	−2.63	184
		Transitional	1.51	0.52	−596
		Superficial	3.14	0.86	1044

The automated approach provided mean T2 value for subregions that contained a lesion of 29.1 ± 4.0 ms, and, for subregions without a lesion, a mean T2 of 27.7 ± 2.7 ms (*P* = 0.133). Volume and thickness were lower in subregions with lesions, 6253 ± 1647 voxels versus 7028 ± 1662 voxels (*P* = 0.142), and 1.92 ± 0.26 mm versus 2.01 ± 0.36 mm (*P* = 0.403). Interestingly, some GLCM features were capable of detecting the subregions that contained a lesion, specifically homogeneity and dissimilarity (*P* = 0.029 and *P* = 0.043, respectively). All values are listed in **Table 4**.

**Table 4.** Mean Values for T2, Volume, Thickness, and Gray-Level Co-Occurrence Matrix (GLCM) Features for Subregions that Contained Lesions and Subregions Without a Lesion.

			*n*	Mean	SD	95% Confidence Interval for Mean		Significance
						Lower Bound	Upper Bound	
	Mean T2 (bulk)	No lesion	60	27.7	2.7	27.0	28.4	0.133
		Lesion	12	29.1	4.0	26.5	31.7	
	Mean T2 (superficial)	No lesion	60	33.9	3.8	32.8	35	0.244
		Lesion	12	35.5	4.0	34.4	36.6	
	Mean T2 (transitional)	No lesion	60	26.8	2.2	26	27.6	0.180
		Lesion	12	27.2	3.4	26.4	28	
	Mean T2 (deep)	No lesion	60	22.3	4.8	21.6	23	0.098
		Lesion	12	24.7	6.7	24	25.4	
Volumetric measures	Voxels	No lesion	60	7028	1662	5972	8084	0.142
		Lesion	12	6253	1647	5828	6679	
	Thickness	No lesion	60	2.012	0.362	1.918	2.105	0.403
		Lesion	12	1.919	0.261	1.754	2.085	
Texture analysis using GLCM	Autocorrelation	No lesion	60	18.29	2.58	17.63	18.96	0.102
		Lesion	12	19.89	4.87	16.80	22.99	
	Contrast	No lesion	60	0.838	0.317	0.756	0.919	0.093
		Lesion	12	0.666	0.330	0.456	0.875	
	Correlation	No lesion	60	0.768	0.062	0.751	0.784	0.118
		Lesion	12	0.798	0.057	0.762	0.834	
	Dissimilarity	No lesion	60	0.567	0.126	0.534	0.599	0.043*
		Lesion	12	0.483	0.137	0.396	0.570	
	Energy	No lesion	60	0.130	0.030	0.122	0.137	0.080
		Lesion	12	0.150	0.060	0.112	0.188	
	Entropy	No lesion	60	2.500	0.162	2.458	2.541	0.230
		Lesion	12	2.432	0.241	2.279	2.585	
	Homogeneity	No lesion	60	0.754	0.041	0.743	0.764	0.029*
		Lesion	12	0.783	0.046	0.754	0.812	

* Statistically significant (*P* < 0.05).

## Discussion

In this study, the reproducibility of automated cartilage segmentation for morphologic and quantitative cartilage evaluation was demonstrated. In addition, the results were compared to manually segmented cartilage, as well as manually evaluated T2 maps, and the ability to detect low-grade cartilage lesions was assessed. The Dice coefficients showed very high agreement between manual and automated segmentation (from 0.825 to 0.882), which was even further improved subsequently, when the automated segmentation was postedited (from 0.828 to 0.895). Test-retest of automated cartilage evaluation showed relatively low SDC, in particular for volume, thickness, and T2 values. Even though the reproducibility of texture features was moderate, 2 of these features (dissimilarity and homogeneity) demonstrated the ability to distinguish between healthy cartilage and damaged cartilage.

Articular cartilage can be visualized and interpreted by using magnetic resonance imaging, especially for the assessment of knee OA, but also for focal cartilage lesions. Manual segmentation of articular cartilage from MR images is a challenging and time-consuming task, yet extremely important for longitudinal OA studies. To date, a plethora of studies have been dedicated to the design of automatic algorithms that would accelerate this process. Different strategies were applied to automatically segment the cartilage, including intensity-based,^16^ edge-based,^17^ region-based,^33^ using deformable models,^21^ clustering-based,^18^ graph-based, region based,^20^ and, recently, very popular CNN-based methods.^34,35^ The most important feature of automated cartilage segmentation approaches is their capability to maintain accuracy and reproducibility when applied to images acquired with different sequences or protocols. The algorithm incorporated in this study is based on the segmentation design proposed by Fripp *et al*.,^36^ which obtains automated segmentations of the cartilage by automatically segmenting the bones and extracting the BCIs in the knee using 1.5- and 3-T images for training purposes. The mean Dice coefficient was 0.853 ± 0.023, and, after post-editing of automated segmentation (mostly involving the correction of mis-segmented posterior parts of femoral cartilage), it increased to 0.866 ± 0.029. These numbers are comparable to other previously published methods, for example, Dodin *et al*^37^. (DSC = 0.85), Yin *et al*^38^. (DSC = 0.84), and Xi *et al*.^39^ (DSC = 0.81). CNN-based methods usually score higher similarity coefficients on chosen datasets. They are, however, trained on a particular dataset with strictly defined image properties (resolution, contrast, signal-to-noise ratio). Moreover, the number of cartilage subregions in CNN-based models is limited to 3 to 5, since a higher number would increase the model complexity enormously. In this study, the reproducibility of cartilage sub-regions was very high for both volume and thickness measurements (*P* = 0.93 and *P* = 0.83, respectively). Cartilage volume and thickness have been used previously as useful biomarkers for the assessment of physiological and pathological effects.^40-42^ The total cartilage volume and thickness alterations reported in these articles was ~10%; thus, the desired reproducibility of any automated approach should be substantially lower to reasonably detect such changes. The mean change in volume and thickness was 1.25% and 1.77% in the test-retest evaluation, respectively, which suggests its usefulness for detecting subtle changes in the course of OA or for treatment monitoring.

Quantitative MR parameters, such as T1, T2, T1ρ, magnetization transfer, and sodium concentration, are valuable markers for determining the cartilage ultrastructure, and thus, they have attracted the attention of the research community.^43^ T2-mapping is widely used in cartilage research, as it can provide information about the collagen matrix organization and hydration.^44^ Similar to the measurement of cartilage volume, T2 analyses of the whole knee cartilage are relatively rare, and are rather performed regionally, either for focal cartilage lesions or for cartilage repair.^45^ In this study, the automated T2 analysis was performed by combining automated cartilage segmentation from morphological images with coregistration of T2 maps onto morphological images. In addition, the cartilage was divided into 3 layers: superficial, transitional, and deep in thirds. Although this does not correspond to the anatomical cartilage structure, where the superficial zone is in the range of a few tens of micrometers, it still makes sense to divide the cartilage into subsegments, since OA—and possibly disease-modifying drugs—may affect the respective cartilage layers differently.

In our study, T2 maps were also evaluated by texture analysis using GLCM. GLCM features have been shown to correlate with OA progression in postmenopausal women,^46^ in patients with diabetes mellitus,^47^ and in patients after anterior cruciate ligament tear.^48^ Comprehensive analyses of texture features suitable for articular cartilage are discussed in an article by Peuna *et al*.^28^ The reproducibility of individual GLCM features, calculated from automatically segmented maps, was lower compared with volume, thickness, and T2, but still acceptable. This can be attributed to the fact that a slightly mis-segmented ROI (typically capturing synovial fluid) does not impact volume, thickness, or T2 substantially; however, texture features could be dramatically altered. From all GLCM features, autocorrelation, dissimilarity, and homogeneity stand out in terms of reproducibility. Moreover, the sensitivity to cartilage degeneration was superior to all other parameters, especially dissimilarity and homogeneity, which were capable of significantly distinguishing healthy cartilage tissue from degenerated tissue. This was only partially in agreement with previously published results of texture analysis of OA-affected cartilage. Williams *et al*.^48^ found contrast, homogeneity, and energy to be the most suitable GLCM features to identify patients with OA. Chanchek *et al*.^47^ used data from the Osteoarthritis Initiative (OAI) to show that, in addition to T2 values, entropy, contrast, and variance were also able to distinguish between volunteers and patients with OA. Our study suggests that using smaller cartilage segments may be beneficial for GLCM analysis, as it introduces smaller errors due to ROI preprocessing (cartilage flattening in particular) and takes into account the natural texture variability in cartilage subregions.

This study has some limitations. The number of scanned and post-processed patients was relatively small. However, considering the 21 cartilage subregions, we believe that sufficient data were available for reliable statistics. Furthermore, only DESS and TESS pulse sequences were tested for automated evaluation, using DESS for morphological imaging and TESS for quantitative T2-mapping. However, in theory, any other isotropic morphological sequence could be used for automated segmentation and any quantitative MR method that provides sufficient contrast could be coregistered with DESS, using the proposed method. This would be highly beneficial in multicenter, large-cohort patient OA trials designed to demonstrate the treatment effect both on cartilage volume and quality. The manual segmentation was not performed in the same 21 cartilage subregions, as it was extremely difficult to reproduce the division provided by automated software. Nevertheless, the automated software could repeat the subregion selection with very high reproducibility. The repeatability of zonal T2 evaluation, as well as the comparison to manual evaluation, was acceptable. However, due to the very low pixel number in each sub-region/layer, the variation was higher than that in bulk analysis. Furthermore, the interpretation of some GLCM features in cartilage texture is unclear. Only a few of these features were assessed in previous studies,^27,28,46,48^ so that a deeper understanding of the GLCM features has yet to be developed.

## Conclusion

The proposed technique of automated knee cartilage evaluation using morphological images provides highly reproducible results and greatly reduces the segmentation effort required for the analysis of knee articular cartilage in longitudinal, large-cohort trials. The 21 cartilage subregions examined offer the possibility of a unique analysis of the whole joint, which allows a more specific analysis of the cartilage with regard to the site of degeneration or the treatment monitoring. In addition, the automated detection of these precisely defined cartilage subregions is a unique procedure that makes this approach particularly useful for studies in patients with knee osteoarthritis, where the cartilage may be degenerated in several areas. Last, the possibility of extracting information from T2 maps about early changes in cartilage texture in these same regions opens a new development path toward qualitative biomarkers for better differentiation of treatment options.
